# Temporary Vascular Occlusion by Rapid Reverse Phase Polymer: A Preliminary In Vitro Study of Retrograde Injection

**DOI:** 10.1155/2012/152845

**Published:** 2012-07-25

**Authors:** Einar Dregelid

**Affiliations:** ^1^Department of Vascular Surgery, Haukeland University Hospital, Jonas Lies vei 65, 5021 Bergen, Norway; ^2^Institute for Surgical Sciences, University of Bergen, Jonas Lies vei 65, 5021 Bergen, Norway

## Abstract

During vascular surgical operations, there is a need for a simpler and more reliable method of temporary arterial occlusion than those currently employed, especially of heavily calcified arteries. A thermosensitive polymer, LeGoo (LG) (Pluromed, Woburn, MA), has been used successfully for temporary vascular occlusion. It has hitherto been injected by a cannula that has been introduced into the artery to be occluded, here henceforth called the “cannulation method.” Injection into arterial ostia without cannulation, using an injection device that arrests blood flow during the injection, here henceforth called “a retrograde method” may enable temporary hemostasis when ostial stenoses render it impossible to inject LG using the cannulation method. The objective of the present study was to study the feasibility of a retrograde method and to compare it with the cannulation method in an in vitro model, incorporating a narrow orifice to simulate ostial stenosis, using tap water at 37°C instead of blood. The retrograde method of LG injection, using a modified paediatric Foley catheter, turned out to be feasible to produce a durable LG plug more reliably, at higher water pressure and with less deep LG injection than with the cannulation method.

## 1. Introduction

For hemostasis during vascular surgical operations, vascular branches need to be temporarily occluded. This may be accomplished by free dissection and clamping of the branches, which add time and operative trauma to the operation, or by occluding balloons introduced into branch ostia for, for example, visceral and large segmental arteries and stay sutures for small segmental ones, both of which clutters the operative field. A simpler yet rapid and reliable method of temporary arterial occlusion is therefore needed. The more rapidly and reliably bleeding from arterial ostia can be stopped during open aortic and other vascular operations, the less untoward blood loss. 

LeGoo (LG) (Pluromed, Woburn, MA, USA) is a thermosensitive polymer (20 weight percent of purified poloxamer 407 in saline) that undergoes rapid transition from liquid to a high viscosity gel when warmed from refrigerator temperature to body temperature and has been used successfully for temporary vascular occlusion in, for example, coronary artery bypass surgery. When used for temporary coronary artery occlusion, LG has been injected by a cannula that has been introduced via the arteriotomy into the artery proximally and distally to the arteriotomy, henceforth called the “cannulation method”. Intra-arterial pressure and flow rate have been reduced during LG injection by compression of the artery by a finger or a small surgical swab [1–3].

When the task is to temporarily stop bleeding from arterial ostia, for example during aortic surgery, digital compression of the artery to reduce intra-arterial pressure and flow rate during LG injection may not be possible, and the cannulation method therefore may not be applicable. It may, however, be possible to inject LG into the ostium without cannulating it using an injection device with, for example, a balloon to seal the connection between the device and the ostium, henceforth called “a retrograde method”. Using this method blood flow will be arrested during the injection. A retrograde method may be applicable when arteries are so calcified that hemostasis cannot be obtained either by application of vascular clamps or intravascular occlusion balloons and when obstructing calcified plaques render it impossible to inject LG using the cannulation method. 

LG does not seem to cause permanent arterial occlusion [1–8] but the return of flow may be delayed by an LG plug that dissolves slowly because it is excessively long or because it is inaccessible to dissolution by cooling [9, 10]. It may be important that LG is not injected so far as to cause side-branch embolization. The risk of this occurring will be smaller, the shorter distance from the ostium LG is injected into a vessel [9]. A retrograde method may possibly enable plug formation without injecting LG as far into the vessel as is needed using the cannulation method.

The objectives of this study was to assess in an in vitro model whether a retrograde method of LG injection may be a feasible method of obtaining temporary hemostasis, whether it may enable temporary hemostasis with LG being injected for a shorter distance into a vessel than with the cannulation method, and whether temporary hemostasis may be obtained more reliably than when the cannulation method is used. 

## 2. Material and Methods

In a series of experiments, the feasibility of temporarily stopping flow of body-warm tap water from a polyvinyl chloride tube using LG was examined with both the retrograde and the cannulation method. For the retrograde method, a modified pediatric Foley catheter was used. The retrograde method was compared with the cannulation method under a number of experimental conditions to see which method most reliably produced a durable LG plug. 

**Figure 1:**
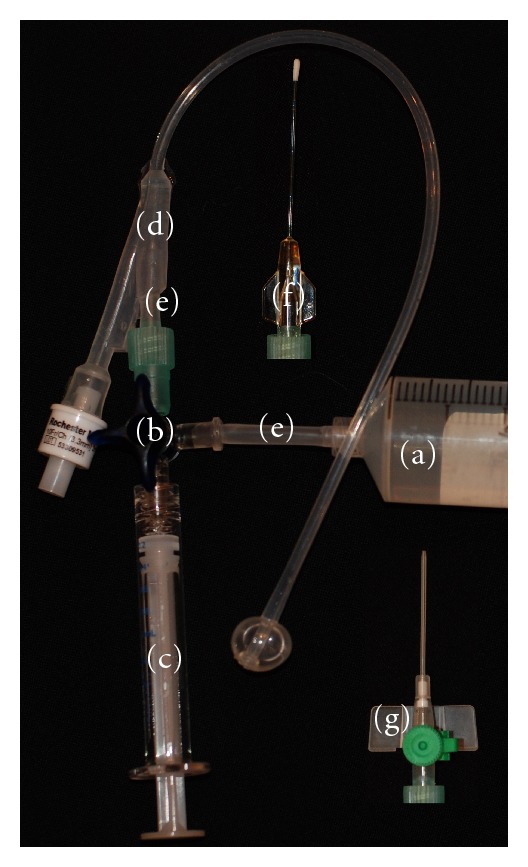
Fifty mL syringe containing LG (a), 3-way stopcock (b), syringe for injection (c), modified paediatric Foley catheter (d), polyethylene tubes (e), and inserts show LeGoo cannula (f) and intravenous cannula (g), both connected to 3-way stopcock.

A paediatric size 10 French Foley catheter (Rochester Medical Corporation, Stewartville, MN, USA) was modified by cutting off the portion in front of the balloon and by cutting off sufficiently from the proximal part of the main lumen to accommodate a polyethylene tube with 3.5 mm inner and 4.6 mm outer diameter as a connector to a 3-way stop cock (Discofix, B. Braun Melsungen AG, Melsungen, Germany). The catheter was used for the retrograde method of injection. The LG for injection, contained in a 50 mL syringe, was transferred to the injection syringe via another polyethylene tube and the 3-way stop cock and kept in an iced water bath (temperature 2.5–7°C) between injections. For the cannulation method, an olivary-tipped LeGoo 1.5 mm × 40 mm cannula (Pluromed Inc, Woburn, MA, USA) or a 1.3 mm × 32 mm intravenous cannula (Venflon, Becton Dickinson Infusion Therapy AB, Helsingborg, Sweden) was attached to the 3-way stop cock and used for injection instead of the modified Foley catheter (Figure 1). Resistance of flow through the olivary-tipped LeGoo 1.5 mm × 40 mm cannula and the 1.3 mm × 32 mm intravenous cannula was tested by timed aspiration of 6 mL of cold water to vacuum in a 10 mL syringe. 

**Figure 2:**
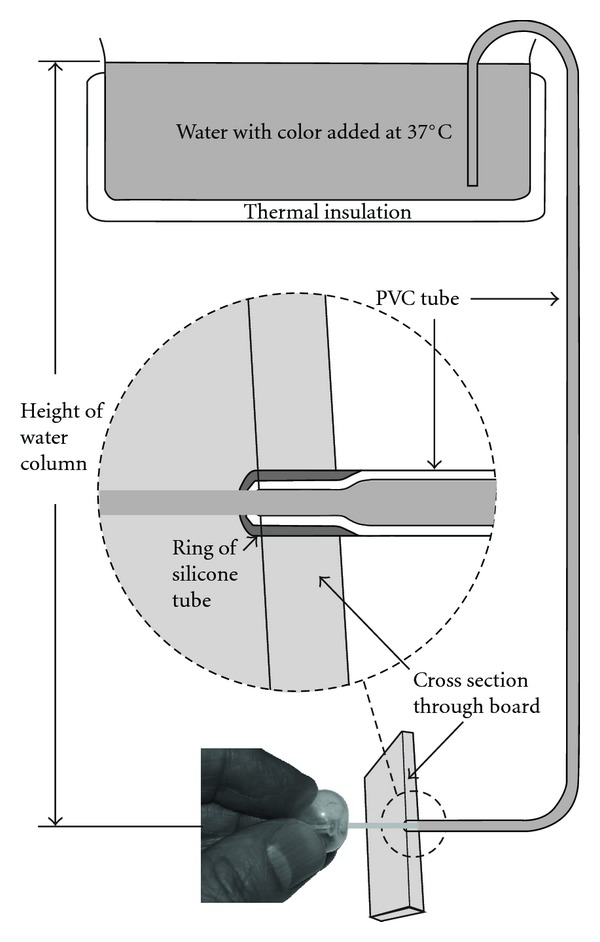
Experimental setup. The PVC tube can be opened and closed using a clamp (not shown). In the depiction, the balloon of the modified Foley catheter is held in front of the orifice while tap water spouts out. The balloon is ready to be pressed against the orifice.

A polyvinyl chloride (PVC) intravenous tube (CODAN pvb Medical GmbH, Lensahn, Germany) of 4 mm outer diameter, 0.5 mm wall thickness, and 7.07 mm^2^ luminal area with a narrowing at its orifice was perfused, using gravity, from a reservoir of tap water at 37°C (34.1–42.8°C) with food colour added (Figure 2). The tube passed through a circular hole (diameter 4.7 mm) in a board (board thickness: 7.3 mm). A ring of silicon tube (inner diameter 3.45 mm, outer diameter 5.4 mm) made the connection between the tube and the board watertight and narrowed the tube at its end. The tube could be opened and closed using a clamp. Water pressure in the tube was determined by the height of the water column. In some experiments, a sponge around the horizontal part of the tube was irrigated with water at a temperature between 35.4°C and 44°C. 

Water flow rate was determined by timed collection of water in a beaker, and flow velocity in the tube (*v*) was calculated by dividing flow rate by lumen area (*A*). Water velocity through the ostium (*v*_*o*_) and effective orifice area (*A*_*o*_) were calculated by measuring the downward deflection of the water jet (*s*) over a horizontal distance (*l*) and applying (1)-(2) as follows:

(1) s=0.5gt2

*g*  = gravitational acceleration (approximately 9.81 ms^−2^),

(2) vo=lt,voAo=vA.

The luminal area of the tube orifice was 3.19 mm^2^, hence there was a 55% reduction in luminal area, and olivary-tipped LeGoo cannulas thicker than 1.5 mm were too thick to be used in this experimental setup. At a water pressure of 91 mmHg, water velocity in the tube was 0.8 m/s, flow rate was 5.7 mL/s, and at 30 mmHg, water velocity was 0.42 m/s, and flow rate was 2.97 mL/s. 

**Figure 3:**
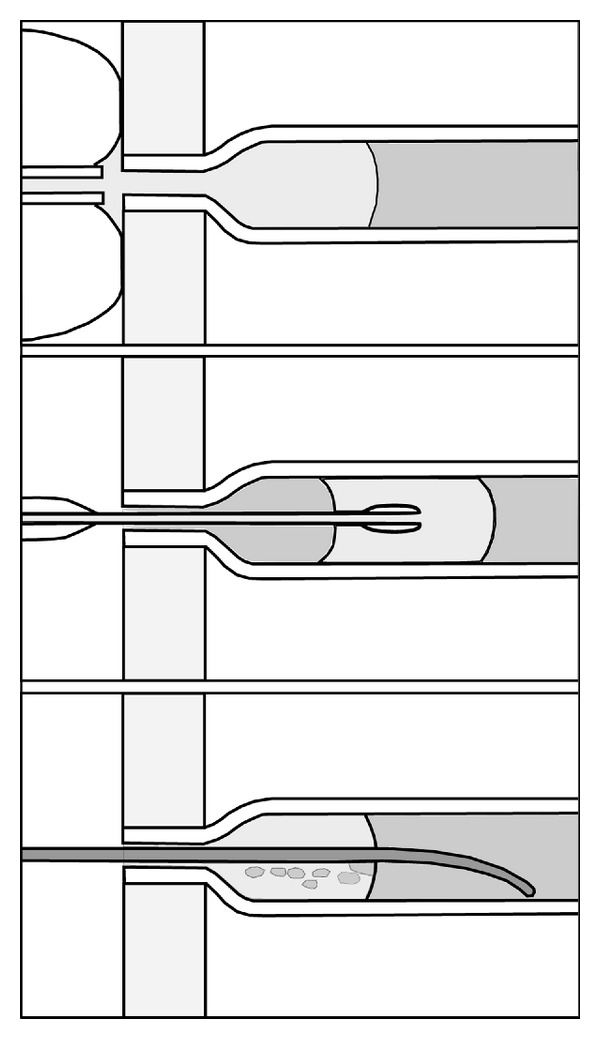
Principle diagram. The upper panel shows retrograde LG injection (or LG plug removal by aspiration) by the modified paediatric Foley catheter. The balloon of the modified catheter is positioned over the tube orifice. The middle panel shows LG injection using the LG cannula. The lower panel shows disruption of an LG plug by a guide wire. LG is shaded with light grey and tap water darker grey.

Under the experimental conditions of each experiment (Table 1), it was recorded whether an occluding LG plug was obtained. The plug's length, any peculiarities about its appearance, and time till flow resumed were noted. Most experiments were made with one selected experimental parameter in mind and, given the exploratory and preliminary nature of the study, not all parameters were noted during each experiment. Plug duration of at least 3 minutes was used as a threshold value for successful plug formation. Prior to each experiment, the plug from a previous experiment that resulted in successful plug formation was removed until coloured water seemed to fill the tube by flushing the surrounding tube with water of 14.5 °C, by inserting a 0.9 mm guide wire through the orifice and moving it repeatedly through the plug (Figure 3, lower panel), or by aspiration over the orifice with a syringe or modified Foley catheter (Figure 3, upper panel). Since a number of plugs appeared to be longer than calculated from the LG volume injected, excess plug volume was calculated as the difference between plug volume and injected LG volume. 

Results are given as median with range in parenthesis. The Fischer-Irvine 2-sided exact test was used for comparison of frequencies and the Wilcoxon two-sample test was used for comparison of two groups. *P* < 0.05 was considered significant. Epi Info 7 was used for statistical calculations. 

## 3. Results

Fifty-two of 104 LG injections produced LG plugs that lasted at least 3 minutes. Most plugs that were observed for >5 minutes were stable and did not change for the next 10–15 minutes.  Table 2 shows the effect of selected experimental parameters on success of plug formation. With the retrograde method, successful plugs were produced in 27 of 53 of injections when water pressure was 91 mmHg and in 10 of 10 injections when water pressure was 30 mmHg, *P* < 0.004. With 21°C water in the balloon, gelatinous LG was pressed out between the balloon and the board (Figures 2 and 3, upper panel) rather than being pressed into the orifice in at least 3 of 5 unsuccessful injections using the retrograde method. Some plugs lasting less than 3 minutes had admixture of some air bubbles. 

With the retrograde method, the use of a 1 mL syringe produced successful plugs in 12 of 14 injections and the use of a 0.5 mL syringe produced successful plugs in 11 of 30 injections, *P* < 0.004. An estimated median injection time of 0.32 s produced successful plugs in 19 of 42 injections, while an estimated median injection time of 0.64 s produced successful plugs in 8 of 11 injections, *P* = 0.175. Although not a significant difference, there is a trend that slower injections produced successful plugs more frequently. 

Using the cannulation method and a water pressure of 30 mmHg, with the 1.3 × 32 mm intravenous cannula, successful plugs were produced in 11 of 13 injections, while with the 1.5 × 40 mm LG cannula successful plugs were produced in 3 of 24 injections, *P* < 0.00003. There was water distally to one of the three successful plugs as well as to some plugs lasting less than 3 minutes showing that the cannula had been inserted unnecessarily far into the tube. 

With the cannulation method, it appeared that LG could not be injected fast enough using hand injection at 91 mmHg perfusion pressure, water flow velocity 0.8 m/s using the 1.5 mm × 40 mm olivary-tipped LG cannula. To compare resistance through the cannulas, 6 mL of cold water was aspirated through the cannulas to vacuum in a 10 mL syringe. It took 5.4 s to aspirate 6 mL of cold water to vacuum through the olivary-tipped 1.5 mm × 40 mm LeGoo cannula and 0.5 s to aspirate the same volume through the 1.3 mm × 32 mm intravenous cannula. 

**Figure 4:**
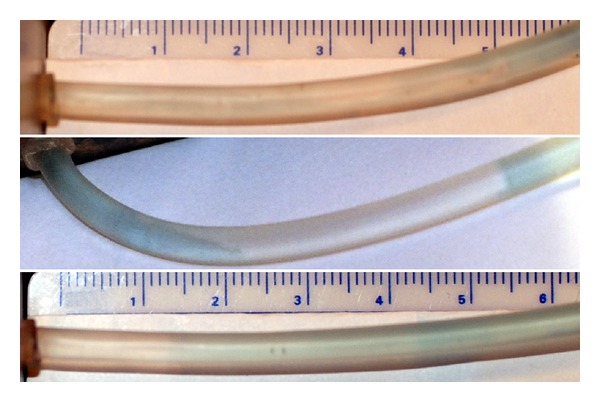
Terminal tube at the board containing coloured water and uncoloured LG. The upper panel shows a 55 mm plug after injection of 0.3 mL LG with the cannulation method using the LeGoo cannula which was retracted during injection. There are traces of water in the plug corresponding to the path that formed on cannula retraction, and the border between water and LG plug is indistinct. This plug lasted only one minute. The middle panel shows a 32 mm plug after injection of 0.2 mL LG through a LeGoo cannula without retraction during injection. There is water on both sides of the plug showing that the plug had formed unnecessarily deep into the tube. The lower panel shows a 33 mm plug after retrograde injection of 0.15 mL LG. The plugs in the middle and lower panels were observed for 20 minutes without observing any change.

When the olivary-tipped cannula was withdrawn after plug formation, in some experiments the olive at the tip of the cannula seemed to effect a drag on the plug, causing the plug to be withdrawn a few millimeters, partially disrupting and making a path in the plug that filled with water (Figure 4, upper panel). This usually led to gradual resumption of flow. Also, in most experiments when the LeGoo cannula was used for injection, the boundaries between the LG plug and water were indistinct.

None of the other parameters examined, volume of LG injected, heating of LG shortly before injection, external heating of tube with water, balloon content and temperature, time from beginning of LG injection till removal of balloon (3.0–5.6 s versus 5.8–10 s), or retraction of cannula during LG injection were shown to significantly determine whether a plug was produced.

 Plug length in 7 experiments with plug duration <3 minutes, 2.75 cm (2.0–5.0 cm), was not significantly different from that of 29 experiments with plug duration >3 minutes, 3.45 cm (0.9–8.0 cm), *P* = 0.19. There was a trend of less excess plug volume, −0.006 mL (−0.059–0.055 mL) in 7 experiments with plug duration <3 minutes, than in 29 experiments with plug duration >3 minutes, 0.055 mL (−0.137–0.266 mL), *P* = 0.055.

 When LG was injected immediately after being cooled in the ice bath, resistance to injection felt similar to that encountered during water injection. When LG had been in a water bath at 21°C before injection or there was water at 21°C in the balloon, one could feel some resistance to injection, and some injections failed because the higher injection pressure made the catheter disengage from the connection to the syringe or made the handle of the 3-way stop-cock disengage from the rest of the stop cock. LG made the components of the stop cock slippery. When the catheter was held with warm fingers for more than a few seconds, the LG started to gel in the catheter. 

LG plugs could be removed within a couple of minutes by flushing the tube containing the LG plug with water of 14.5°C, within a minute by moving a guide-wire repeatedly through the plug (Figure 3, lower panel) or, immediately, by aspiration over the orifice with a syringe or modified Foley catheter (Figure 3, upper panel). 

## 4. Discussion

The most important finding in this study was that water flow in a PVC tube with a narrow orifice at its end could be arrested more reliably by injecting LG by the retrograde method than by the cannulation method using a LeGoo cannula. In the experimental setup it was elected to use a narrow orifice to simulate the frequently occurring clinical situation of a calcified stenotic arterial orifice that may not allow introduction of thicker olivary cannulas or occlusion balloons. The pressure values, 91 and 30 mmHg, were chosen to simulate high and low collateral back pressure. At high water pressure and flow rate through the tube, water stasis could only be obtained with the retrograde method and not with the cannulation method using the LeGoo cannula. LG flow rate through the high-resistance LeGoo cannula was probably lower than water flow rate through the tube in most experiments, prohibiting plug formation with manual injection. At low pressure and flow rate, cannula resistance may still have played a role, although pulling the olive through the plug, at least in some experiments, seemed to disrupt and move the plug slightly, at least contributing to early plug dissolution. 

The lack of success with the cannulation method for LG injection using the LeGoo cannula in this in vitro study is in contrast to the success seen in studies with temporary coronary occlusion with LG during coronary artery bypass [1, 10]. In those studies, however, coronary artery pressure and flow at the site of injection was reduced during LG injection, probably below 30 mmHg, by compression of the coronary artery with a finger or small surgical swab. In the current study variable partial obstruction of the orifice by the proximal wider part of the cannulas may also have retarded water flow and facilitated plug formation. If coronary arteries and perivascular tissues have higher heat capacity and thermal conductivity than that of the PVC tube in the current in vitro study, it could also contribute to explain the discrepancy. 

With the retrograde method, more successful plugs were produced using a 1 mL syringe than with a 0.5 mL syringe. Since a large syringe produces more resistance to injection than a small one, injection velocity may have been slower with the 1 mL syringe than with the 0.5 mL syringe, syringe size most likely having been a proxy for injection velocity. Using the retrograde method with the selected catheter dimension, there may be an optimum injection velocity with injections using the 0.5 mL syringe being too rapid and injections using the 2.5 mL syringe possibly being too slow. The attempt to standardize injection velocities by counting to 1 or 2 during injection did not result in uniform injection velocities within each injection velocity group (Table 1) explaining the lack of a significant effect of this experimental parameter on success of plug formation. 

In the current study, tap water was used instead of blood. Viscosity of blood and water are similar (approximately 0.004 and 0.0007 Pa s, resp., at 37°C), orders of magnitude lower than that of LG (approximately 1500 Pa s at 37°C) [8, 11, 12]. Therefore, resistance during injection is dominated by the viscosity of LG as it is warmed up during injection. During retrograde injection, ice-cooled LG appeared to retain a sufficiently low viscosity throughout the injection so that no increase in resistance could be felt. With LG at 21°C, increasing viscosity, hence higher resistance during injection as LG was warmed up further explains why some injections failed.

While suboptimal injection velocity in part may explain why retrograde injections did not produce successful plugs in 49% of injections, none of the other parameters examined, volume of LG injected, heating of LG shortly before injection, external heating of tube with water, balloon content and temperature, time from beginning of LG injection till removal of balloon, or retraction of cannula during LG injection were shown to explain the binary outcome of successful plug formation, but this preliminary study was not sufficiently powered to show an influence of these parameters.

One hypothetical, unstudied cause of lack of success with the retrograde method is that the plug in the tube, being in continuity with the gel in front of the balloon catheter may have been retracted by removing the latter backwards instead of laterally which may shear the plug without pulling on it. Another hypothetical cause is that water admixture from the ice-water bath to the LG at the end of the catheter may have occurred.

With the retrograde method using the modified balloon catheter it is necessary to press the latter against the arterial wall at the site of the orifice through which LG is to be injected. This will be possible when the arterial wall is stiff, for example, due to calcification or fibrosis. Whether the method is applicable when the arterial wall and surrounding tissues are soft and yielding needs to be ascertained in further studies. Possibly, some of the available coronary cannulas may be better suited when the artery around the orifice is soft and pliable.

LG has been used for temporary coronary artery occlusion without evidence of detrimental effects due to possible embolization to side branches. In the heart, the massaging effect on the coronary arteries by the moving myocardium may help to dissolve and get rid of remaining LG. The safety with regard to side branch embolization has not been similarly well documented in other organs. Avoidance of side-branch embolization may therefore be important and is most likely to be avoided by injecting only the least necessary LG volume starting at the orifice to obtain temporary hemostasis. With the cannulation method using the LeGoo cannula there is a risk that the cannula may be inserted further than necessary into the vessel (Figures 3 and 4, middle panels) with attendant unnecessary risk of embolization and prolonged occlusion. Using the retrograde method, the LG plug always extends from the ostium (Figure 3, upper panel and Figure 4, lower panel).

The instructions accompanying LeGoo as well as some authors recommend retraction of the cannula during LG injection to avoid uneven coating of the gel [10, 13]. Other rationales could be more rapid warming of LG by injecting along a stretch of vessel wall and prevention of path formation and partial plug disruption due to retraction of the olive through the plug. In the experimental setup used in this study, however, no advantage was seen by retraction of the cannula during injection although the optimal speed of cannula retraction in relation to injection and blood velocity has not been defined, and retraction velocities may not have been optimal in the current study.

The use of an olivary cannula may reduce the risk of intimal injury and, since an olive causes some obstruction and reduces blood flow rate, plug formation may be facilitated compared with a cannula without an olive. The present in vitro study suggests that these possible advantages may be offset by the greater resistance during injection and the disruptive effect on the plug of the olivary cannula. 

Although all visible LG was removed after each injection, the presence of positive excess plug volumes show that some LG was present on the interior tube wall after the previous experiment. The trend of less excess plug volume when plug duration was <3 minutes than when plug duration was >3 minutes suggest more successful plug formation when some LG was present in the tube after the previous experiment than when it was not present. 

## 5. Conclusions

A “retrograde method” of injection of thermosensitive polymers into arterial ostia without cannulation, using an injection device that arrests blood flow during the injection, is feasible.

It can produce more pressure-resistant plugs more reliably and by less deep LG injection, hence with less risk of embolization and prolonged occlusion than the cannulation method with the LeGoo cannula. The olive of olivary cannulas may prevent the entrance of sufficiently thick cannulas through arterial stenoses and it tends to disrupt and move the plug during retraction contributing to early plug dissolution. 

## Figures and Tables

**Table 1:** Experimental parameters.

Relating to all injection methods (retrograde and cannulation with LeGoo cannula and with intravenous cannula)
Water pressure: 30 or 91 mmHg
External heating of tube with water at temperature between 35.4^°^C and 44^°^C: yes or no
Warming LG shortly in water bath of 21^°^C before injection: yes or no
Estimated median LG injection time: 0.32 s or 0.64 s*
Volume of LG injected: 0.15, 0.20, 0.30, 0.40, or 0.50 mL
Volume of syringe for LG injection: 0.5, 1, or 2.5 mL

Relating to the retrograde method
Balloon content and temperature: air, 21^°^C; water, 4^°^C; water, 21^°^C
Time from beginning of LG injection till removal of balloon: short, 3.0–5.6 s or long, 5.8–10 s

Relating to the cannulation method
Retraction of cannula during LG injection: yes or no

^∗^LG was injected while counting to 1 or to 2. In separate experiments, time elapsed was measured while injecting water and counting to 1 or to 2 at the same pace as during LG injection. Time elapsed while counting to 1 was 0.32 s (0.28–0.67 s) and time elapsed while counting to 2 was 0.64 s (0.33–0.77 s).

**Table 2:** Effect of injection method, water pressure, estimated LG injection time, and syringe volume on the number of experiments with successful/unsuccessful LG plug formation^∗^.

Injection method	Water pressure (mmHg)	Estimated LG injection time (s)	Plug formation successful (yes/no)	Syringe volume (mL)	Plug formation successful (yes/no)
Cannulation with LeGoo cannula	30			0.5	3/16
	30	0.32	0/9	1.0	0/5
	91	0.32	0/1	1.0	0/1

Cannulation with i.v. cannula	30			0.5	10/1
	30	0.32	10/2	1.0	1/1
	91	0.32	1/2	0.5	1/2

Retrograde injection using modified Foley catheter	30	0.32	3/0	0.5	10/0
	91	0.32	19/23		
	91	0.64	8/3		
	91			0.5	11/19
	91			1.0	12/2
	91			2.5	4/5

LG: LeGoo; ^∗^successful plug formation denotes duration of plug >3 minutes; i.v.: intravenous.
